# Reduced stability of mRNA secondary structure near the translation-initiation site in dsDNA viruses

**DOI:** 10.1186/1471-2148-11-59

**Published:** 2011-03-07

**Authors:** Tong Zhou, Claus O Wilke

**Affiliations:** 1 Section of Pulmonary, Critical Care, Sleep and Allergy, Department of Medicine, and Institute for Personalized Respiratory Medicine, University of Illinois at Chicago, Chicago, IL 60612, USA; 2 Center for Computational Biology and Bioinformatics, Institute for Cell and Molecular Biology, and Section of Integrative Biology, The University of Texas at Austin, Austin, TX 78712, USA

## Abstract

**Background:**

Recent studies have demonstrated a selection pressure for reduced mRNA secondary-structure stability near the start codon of coding sequences. This selection pressure can be observed in bacteria, archaea, and eukaryotes, and is likely caused by the requirement of efficient translation initiation in cellular organism.

**Results:**

Here, we surveyed the complete genomes of 650 dsDNA virus strains for signals of reduced stability of mRNA secondary structure near the start codon. Our analysis included viruses infecting eukaryotic, prokaryotic, and archaeic hosts. We found that many viruses showed evidence for reduced mRNA secondary-structure stability near the start codon. The effect was most pronounced in viruses infecting prokaryotes, but was also observed in viruses infecting eukaryotes and archaea. The reduction in stability generally increased with increasing genomic GC content. For bacteriophage, the reduction was correlated with a corresponding reduction of stability in the phage hosts.

**Conclusions:**

We conclude that reduced stability of the mRNA secondary structure near the start codon is a common feature for dsDNA viruses, likely driven by the same selective pressures that cause it in cellular organisms.

## Background

Translation initiation is facilitated by specific nucleotide patterns near the start codon. Upstream of the start codon, sequence features such as the Shine-Dalgarno sequence (in prokaryotes) and the Kozak sequence (in eukaryotes) prime the ribosome to initiate translation [1-7]. Downstream of the start codon, various sequence features promote translation initiation. For example, in *Escherichia coli*, the codon AAA seems to enhance translation initiation [8]. More generally, translation initiation is enhanced if the mRNA downstream of the start codon is AT-rich and does not form a stable secondary structure [9-13].

Experimental and computational work in *E. coli *showed that gene expression levels are correlated to the thermodynamic stability of mRNA secondary structure near the start codon—lower stability implied higher protein abundance [13]. Recent computational studies have shown that the secondary-structure stability of mRNA segments near the start codon is on average lower than expected [14,15]. Tuller et al. found that, in both *E. coli *and *Saccharomyces cerevisiae*, mRNA secondary-structure stability is reduced at the beginning of ORFs [14]. A more comprehensive study by Gu et al. demonstrated that this reduction in stability occurs in most cellular organisms, including bacteria, archaea, fungi, plants, insects, and fishes [15]. The reduction in stability generally increased with increasing genomic GC content. In birds and mammals, the pattern was not found genome-wide but did occur in the most GC-rich genes.

Here, we extended the analysis of Gu et al. [15] to dsDNA viruses. We analyzed the local mRNA secondary structure at the 5' end of the coding region in 650 dsDNA virus strains. We used computational methods to predict the thermodynamic stability of local mRNA secondary structure in sliding windows downstream from the start codon, as described [15]. We addressed the following questions: (i) Is there a selection pressure on synonymous sites to reduce the stability of local mRNA secondary structure at the translation-initiation region in dsDNA viruses? (ii) Are overlapping open reading frames confounding the results? (iii) Does 5' mRNA stability correlate with GC composition? (iv) Does the selection pressure depend on the kingdom of the host organism? (v) Does the selection pressure correlate with other host properties, such as the host's GC content?

## Results

### Reduced mRNA stability at the translation-initiation region in viral genomes

We performed a sliding-window analysis of mRNA secondary-structure stability in 650 fully sequenced ds-DNA viruses. For each ORF in each virus, we calculated *Z *scores *Z*_Δ*G*_. *Z*_Δ*G *_measures to what extent mRNA secondary-structure stability deviates from random expectation given the amino-acid sequence and codon composition of the ORF [15]. A *Z*_Δ*G *_> 0 indicates that the structure is less stable than expected, and a *Z*_Δ*G *_< 0 indicates the opposite. We calculated *Z*_Δ*G *_> 0 for windows of length 30 nucleotides (nt), and we covered the first 150 nt of each ORF in steps of 10 nt, as described [15]. For each window, we then averaged *Z*_Δ*G *_over all ORFs in each genome. We refer to this genome-wide average as . Below, we test whether this genome-wide average is significantly different from zero. Note that the genome-wide average can be significantly non-zero even if the *Z *scores for individual genes are relatively small and would individually not be considered significant.

**Figure 1:**
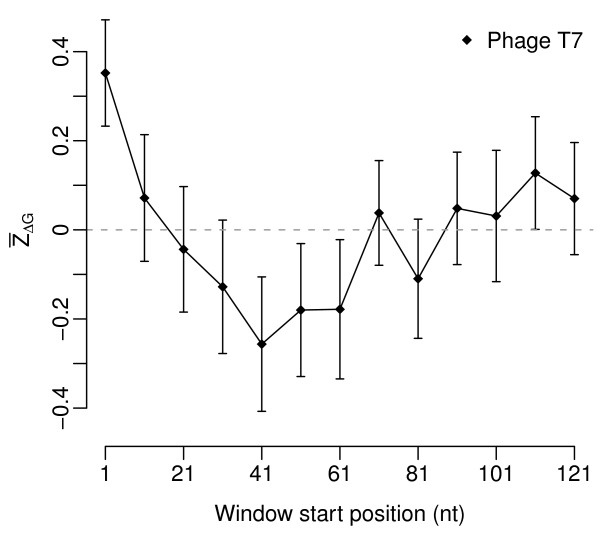
**Genome-wide average  for Enterobacteria phage T7, as a function of the location of the analysis window**. A value of  > 0 indicates less stable mRNA secondary structure than expected. Error bars indicate the standard error. The value of  in the first window is significantly different from zero (*P *= 0.005), the other ones are not.

**Figure 2:**
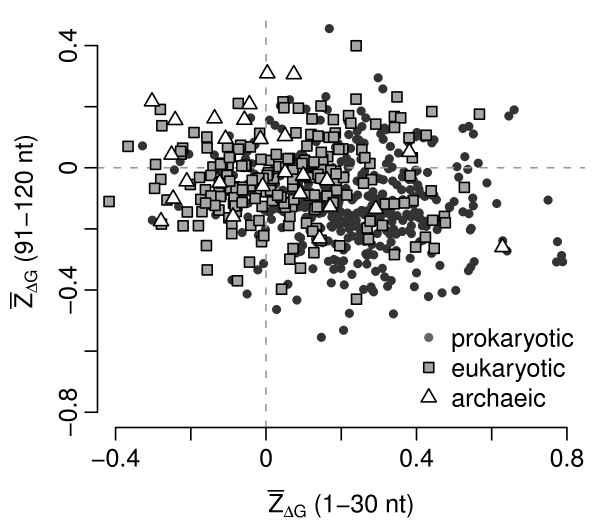
** in the 10th window versus  in the 1st window**. There is a tendency for points to fall into the bottom-right quadrant, indicating decreased mRNA secondary-structure stability near the start codon and increased stability further downstream. However, the effect is much less pronounced than in cellular organisms.

**Figure 3:**
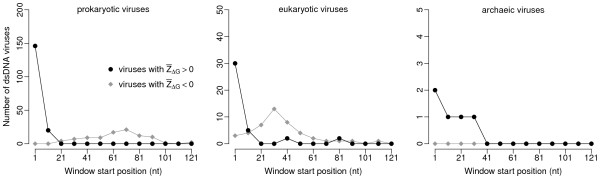
**Number of dsDNA virus strains with significantly non-zero  after false-discovery-rate correction for multiple testing, as a function of the location of the analysis window**. The analysis was carried out on 419 dsDNA viruses with prokaryotic hosts, 204 with eukaryotic hosts, and 27 with archaeic hosts. A value of  > 0 indicates less stable mRNA secondary structure than expected.

Figure 1 shows  as a function of window position for bacteriophage T7. In this virus,  is significantly larger than zero in the first window (*t*-test,  = 0.35, *P *= 0.005), and it is not significantly different from zero in windows further downstream. We carried out a similar analysis on all virus strains in our data set. As in cellular organisms, there was substantial variation in the  of the first window among different virus strains and somewhat less variation in windows further downstream (Figure 2). Allowing for a false-discovery rate of 5%, we found 181 dsDNA viruses (28%) whose  in the first window was significantly non-zero. With the exception of 3 viruses infecting eukaryotes (epiphyas postvittana nucleopolyhedrovirus, cowpox virus, and canarypox virus),  was positive in all these cases. For windows further downstream, the number of virus strains with significantly non-zero  declined rapidly, and  tended to be negative rather than positive (Figures 2 and 3). These results mirror the results of Gu et al. [15], who found that  was generally positive near the start codon and negative further downstream. The main difference in the virus data set is that virus genomes tend to be small, and the error estimates on  are consequently large. (E.g., compare Figure 1 of the present study to Figure 1 of [15].)

In aggregate,  values for eukaryotic and archaeic viruses were lower than those for prokaryotic viruses. On average, archaeic viruses had a  of 0.0049, not significantly different from zero (*t*-test, *P *= 0.91). Eukaryotic viruses had an average of 0.057, which was significantly different from zero (*t*-test, *P *= 3.2 × 10^-5^). Prokaryotic viruses had an average of 0.22, also significantly different from zero (*t*-test, *P *< 10^-10^). The  distribution for prokaryotic viruses was significantly different from those for eukaryotic viruses (*t*-test, *P *< 10^-10^) and archaeic viruses (*t*-test, *P *= 2.1 × 10^-5^).

Since we obtained *Z*_Δ*G *_by shuffling codons within genes, we implicitly assumed that there is no sub-stantial, site-specific selection on synonymous sites outside the focal analysis window. This assumption is violated in regions where reading frames overlap and synonymous sites are primarily determined by the amino-acid sequence of the overlapping reading frame. Therefore, we tested whether our *Z*_Δ*G *_values were confounded by overlapping reading frames. For all ORFs in all virus genomes, we determined whether they overlapped with any other ORFs and classified them into overlapping and non-overlapping ORFs. We found that on average 50% of the ORFs in a virus genome were overlapping, with a standard deviation of 15.9 percentage points. We then tested for each window in each virus genome whether the mean *Z*_Δ*G *_for overlapping genes was different from the mean *Z*_Δ*G *_for non-overlapping genes, using *t *tests. We found that this was generally not the case. Allowing for a false-discovery rate of 5%, not a single virus genome showed a significant difference between overlapping and non-overlapping ORFs in the first four windows. Over all 13 windows, there were only two cases were we could reject the null hypothesis of no difference, invertebrate iridescent virus 3 in window 5 and clanis bilineata nucleopolyhedrosis virus in window 11.

However, when pooling data from all viruses into a single analysis, we found a small shift towards lower  values for overlapping ORFs in eukaryotic and archaeic (but not prokaryotic) viruses (Additional File 1 Figure S1). We concluded that there was no evidence that overlap influences  in prokaryotic viruses, and weak evidence that it does so in the other two types of viruses. Since gene overlap certainly does not increase , we concluded that including both overlapping and non-overlapping ORFs in our analysis was a conservative approach. Therefore, we freely mixed overlapping and non-overlapping ORFs throughout our analysis. The reduced  in eukaryotic and archaeic viruses compared to prokaryotic viruses was not due to the inclusion of overlapping ORFs;  values for all virus groups were nearly identical regardless of whether overlapping ORFs were included or not (not shown).

For a few select virus species, we also tested whether our results were confounded by dinucleotide frequencies. We calculated alternate *Z*_Δ*G *_values using reshuffled sequences in which all dinucleotide frequencies had been held constant. We found that our standard shuffling method and the dinucleotide shuffling method resulted in nearly identical *Z *scores (Additional File 1 Figure S2). Note that in the dinucleotide shuffling method, we shuffled synonymous codons such that both amino-acid sequence and dinucleotide frequencies were held constant. The algorithm to perform this shuffling is fairly computationally expensive and runs approximately 24 times slower than regular codon shuffling.

### Relationship between genomic GC composition and mean 5' *Z*_Δ*G*_

For the remainder of this work, we refer to the  at the very start of the coding sequence (in sliding window #1) as the 5' . To explain the variation observed in the 5' , we correlated it with the mean GC content in coding sequences, since this quantity is a good predictor of the 5'  in cellular organisms [15].

**Figure 4:**
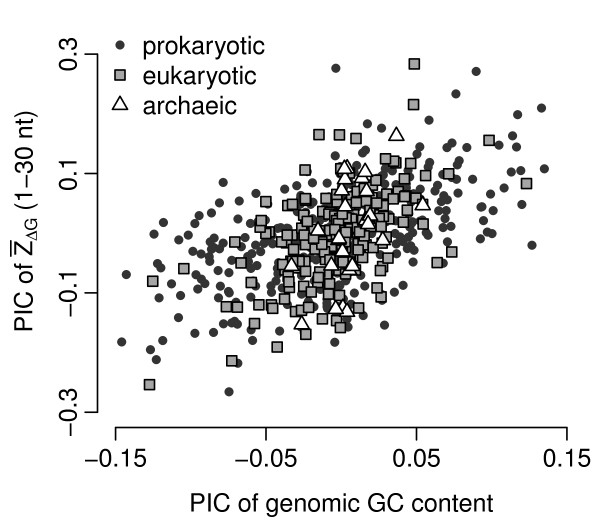
**PIC of  of the first window versus PIC of the viruses' genomic GC content**.

Because different virus strains are evolutionarily related, a relationship between 5'  and GC content may be confounded by the viral phylogeny [16]. We can avoid this issue by correlating phylogenetically independent contrasts (PIC), which are differences of variables among organisms [16]. We found that the PIC of the 5'  were well correlated with the PIC of the GC content in coding sequences (*r *= 0.53, *P *= 10^-31 ^for prokaryotic viruses, *r *= 0.54, *P *= 10^-17 ^for eukaryotic viruses, and *r *= 0.49, *P *= 0.009 for archaeic viruses, see also Figure 4). Genomes with higher GC content had comparatively less stable mRNA secondary structure near the start codon.

Because mRNA stability was reduced only at the translation-initiation region, we expected that the correlation between PIC of  and PIC of GC content should decrease when  was caluclated for windows further downstream. Thus, we calculated the corresponding correlation coefficient for all windows. We found that indeed the correlation declined continuously and was consistently near zero (for eukaryotic viruses) or negative (for prokaryotic or archaeic viruses) from the 5^th ^window onwards (Additional File 1 Figure S3).

Since the thermodynamic stability of RNA secondary structure tends to be correlated to the RNA's GC content, we also considered local deviations in a gene's GC content. We calculated *Z*_GC_, which measures the deviation in GC content in a 30 nt window relative to the average GC content in the gene (see Materials and Methods). We found a negative correlation between PIC of genomic GC content and PIC of  in the first window (*r *= -0.67, *P *= 10^-84 ^for prokaryotic viruses, *r *= -0.68, *P *= 10^-58 ^for eukaryotic viruses, and *r *= -0.74, *P *= 10^-35 ^for archaeic viruses, see also Additional File 1 Figure S4). Thus, in GC-rich viruses, the sequence regions immediately downstream of the start codon have undergone stronger GC reduction.

We also analyzed to what extent Δ*G *(rather than its deviation from expectation, as measured by *Z*_Δ*G*_) varied with GC content. Our results mirrored those we had previously found for cellular organisms [15]. There was a strong negative correlation between the mean Δ*G *and GC content (Additional File 1 Figure S5). The higher the GC content, the more stable the mRNA secondary structure, even in the first window. We also tested for a correlation between GC content and the difference in stability between the first window and the tenth window, and found that this difference increased in viruses infecting prokaryotic or eukaryotic hosts, but not in those infecting archaeic hosts (*r *= 0.68, *P *< 10^-15 ^for prokaryotic viruses, *r *= 0.37, *P *= 10^-7 ^for eukaryotic viruses, and *r *= 0.37, *P *= 0.06 for archaeic viruses, see also Additional File 1 Figure S6). These results remained unchanged when correcting for phylogeny (not shown).

### Host-specific patterns in bacteriophages

Finally, we asked to what extent sequence features in viruses correlated with corresponding features in their hosts. Previous work has shown that synonymous codon usage in bacteriophages exhibits significant bias towards host-preferred codons [17], and that genomic GC content in some phages is close to the genomic GC content of their host organisms [18,19]. Thus, we would expect more generally that both GC content and 5'  in viruses correlate with the same quantities in the appropriate host organisms.

**Figure 5:**
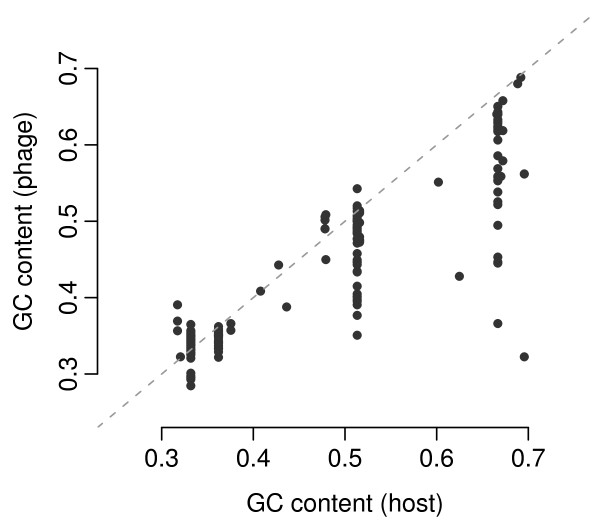
**Mean GC content of coding sequences in bacteriophages versus the same quantity in the associated host organisms**. The dashed line indicates positions of indentical GC content for phages and hosts.

For all bacteriophages in our data set, we identified the corresponding host organism based on the information provided by RefSeq. We then compared GC content in phages and hosts. We found that the GC content in phages correlated strongly with the GC content of the host (correlation coefficient *r *= 0.89, *P *≪ 10^-15^, Figure 5). More specifically, Figure 5 suggests that the phage's GC content places a lower limit on the GC content of host organisms the phages can infect. Nearly all data points fall below the dashed line indicating identical GC content for phages and hosts. Moreover, some phages with low GC content are associated with hosts with high GC content, but phages with high GC content are never associated with hosts with low GC content.

The correlation between phage and host GC content may, however, be confounded by phylogeny, as explained in the previous subsection. One complication here is that the phylogeny of phages is not necessarily the same as the phylogeny of the hosts. We are not aware of any method that can correctly compare two data sets with distinct covariance structures. Therefore, we opted for two strategies. On the one hand, we calculated correlations without considering phylogeny at all, and thus obtained the value *r *= 0.89 cited above. On the other hand, we considered the GC content of the host a measurement on the virus, and thus used the virus phylogeny to calulate PIC for both virus and host GC content. The correlation we obtained in this way was nearly indistinguishable from the one obtained not controlling for phylogeny at all (correlation coefficient *r *= 0.88, *P *= 10^-60^, Additional File 1 Figure S7).

**Figure 6:**
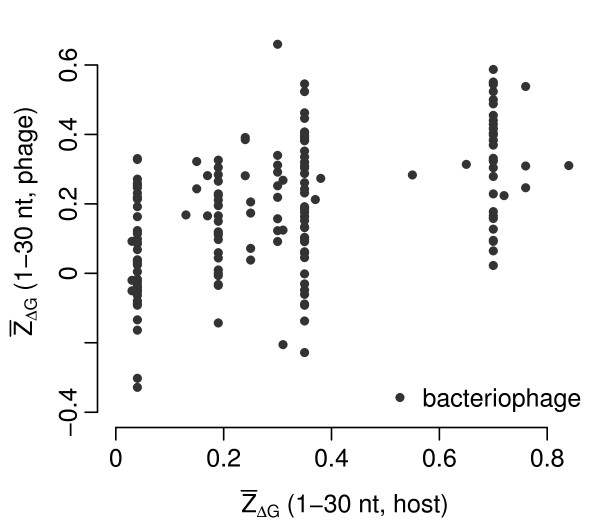
**5'  in bacteriophages versus the same quantity in the associated host organisms**.

We also analyzed whether the 5'  in bacteriophages correlated with that in their hosts. The 5'  values for the phage hosts were obtained from [15]. We found a significant positive correlation, both when ignoring the phylogeny (correlation coefficient *r *= 0.53, *P *≪ 10^-12^, Figure 6) and when using the phage phylogeny to calculate PIC for both phage and host 5'  (correlation coefficient *r *= 0.50, *P *≪ 10^-12^, Additional File 1 Figure S8).

## Discussion

We have studied the mRNA stability at the translation-initiation region of protein-coding genes in 650 genomes of dsDNA viruses. We have found for many of these viruses that there is a tendency for reduced mRNA stability in the first 30-40 nt of the coding sequence. In this region, mRNA stability tends to be less than expected given a gene's amino-acid sequence and codon-usage bias. We have also found that GC content of coding sequences is a major predictor of the reduction in mRNA stability. The higher the GC content, the larger the reduction in mRNA stability at the 5' end of the coding sequence (i.e., the larger 5' ). For bacteriophage, the 5'  also correlates positively with the 5'  in the host organisms.

Experimental and computational work had previously shown that increased local mRNA stability in the translation-initiation region impaired translation initiation in *E. coli *[11,13]. Two computational studies suggested that this effect exists more broadly in both prokaryotes and eukaryotes [14,15]. Here, we have shown that similar selection pressures exist in the viral kingdom.

As in cellular organisms, the region with reduced mRNA stability is located right downstream from the start codon and has a length of 30 to 40 nt (the first two windows in our analysis). Past the first two windows,  tends to be zero or slightly negative. In cellular organisms,  is consistently negative downstream from the start codon [15]. The lack of a negative  in most virus genomes likely reflects lack of statistical power, a consequence of the small genomes of viruses. The strong positive correlation between genomic GC composition and the reduction of mRNA stability at the translation-initiation region is in agreement with the finding by Gu et al. [15].

In contrast to cellular organisms, viruses frequently have overlapping ORFs. In fact, nearly all viruses in our analysis had at least one overlapping ORF. Our codon-shuffling approach conserves the amino-acid sequence of the focal ORF, but does not conserve the amino-acid sequence of any second ORF that overlaps with the focal one. Thus, overlapping sequences will experience additional selective constraint that our approach does not accurately take into account. In principle, this issue could cause spurious results. However, we found that there is little difference in  values in overlapping and non-overlapping ORFs. At worst,  values in overlapping ORFs are reduced compared to those in non-overlapping ORFs (Additional File 1 Figure S1). Therefore, treating overlapping ORFs as non-overlapping ORFs, as we have effectively done throughout much of this work, is a conservative approach when looking for elevated  values.

To understand why  increased with increasing GC content, we also considered the raw Δ*G *values. One can envision two extreme cases of how Δ*G *might depend on GC content. On the one hand, the Δ*G *in the first window might be required to be at a fixed low value, independent of GC content, to enable efficient translation. The Δ*G*s further downstream would be expected to decrease with increasing GC content, due to the higher thermodynamic stabiltiy of GC bonds. On the other hand, the Δ*G *in the first window might always differ by a fixed amount from Δ*G*s further downstream, independent of GC content. We found the reality to be somewhere in between these two extreme cases. Even though the Δ*G *in the first window showed a strong negative correlation with GC content, the difference in Δ*G *was not constant for prokaryotic or eukaryotic viruses, for which it increased strongly and moderately, respectively. For archaeic viruses, however, it did not significantly increase. Since the correlation between  and GC was of comparable magnitude for all three groups, we infer that two separate mechanisms are at play. First, for prokaryotic and eukaryotic viruses, the requirement for decreased stability in the first window increases with increasing GC content. Second, in general, the  measure seems to become more powerful for sequences with increased GC content, because the higher the GC, the less likely it is that a reshuffled sequence shows reduced stability.

For bacteriophages, we addressed the question whether the requirement of low mRNA secondary-structure stability in host genomes affects the 5'  in phages. Because phages share the cellular environment and translation machinery with their hosts, we would expect that phages are optimized for the expression machinery of their hosts. We found a significant positive correlation between the 5'  in phage genomes and that in their hosts. We also observed an even stronger correlation between the genomic GC content in phages and that in their hosts. Moreover, we found that a phage's GC content seems to impose a lower limit on the GC content of the hosts it can infect (Figure 5). These host-specific results are consistent with previous reports that synonymous codon usage in bacteriophage mimicks that of their hosts [17] and that viral and host GC content are similar in certain cases (*Mycobacterium tuberculosis*, 63.6% phage vs. 65.6% host, [18]; *Staphylococcus aureus*, 33.7% phage vs. 32.9% host, [19]).

We used independent contrasts to assess whether  correlated with GC content. The independent contrasts method requires an accurate phylogeny of the organisms under study. Such a phylogeny is difficult to obtain for viruses, because viruses have either arisen multiple times independently or their common ancestor is extremely ancient [20-23]. In our analysis, we separately considered viruses infecting eukaryotes, prokaryotes, and archaea, and used phylogenetic trees derived from the taxonomic classification of these viruses. The branch lengths in these trees reflect simply the number of taxonomic levels that two viruses are separated by. Therefore, the branch lengths are almost certainly incorrect. Nevertheless, these trees should at a minimum remove any major biases that might arise if some groups of viruses were more heavily sampled than others. We found generally that the results based on PIC were nearly identical to results calculated on the raw data (not shown). Therefore, we believe that our results are not strongly confounded by phylogeny and that the correction for phylogeny we employed was sufficient.

In our comparison of viruses with their hosts, we encountered the added complication that virus and host trees will in general not be identical. We are not aware of any method that can calculate correct correlations in this scenario. We addressed this issue by considering both the raw data and PIC based on the virus trees (because properties of the virus host can be considered as a measurement on the virus). Again, both methods produced nearly identical results. Thus, it is unlikely that the results are strongly confounded by phylogeny.

## Conclusions

Many dsDNA viruses show evidence for reduced mRNA secondary-structure stability near the start codon. The effect is the strongest in viruses infecting prokaryotes, but exists also in viruses infecting eukaryotes and archaea. For bacteriophage, the reduction tends to co-occur with a corresponding reduction of stability in the phage hosts. Thus, the same selective pressures that cause reduced stability of mRNA secondary structure in cellular organisms likely also act on the viruses infecting these organisms.

## Methods

We collected virus genomes from the NCBI RefSeq project ftp://ftp.ncbi.nih.gov/refseq/release/viral/. We only considered coding sequences longer than 50 codons. We also excluded virus genomes that had 10 or fewer genes (overlapping reading frames were considered as different genes). We ended up with 650 genomes for dsDNA viruses (419 with prokaryotic hosts, 204 with eukaryotic hosts, and 27 with archaeic hosts). To test whether overlapping ORFs confounded our analysis, we classified all ORFs into overlapping and non-overlapping ones. We defined the ORFs that had no genome region shared with any other ORFs as non-overlapping ORFs. All the other ORFs were considered as overlapping ones.

We analyzed the stability of local mRNA secondary structure exactly as described [15]. In brief, we calculated the local folding energy (Δ*G*) along the mRNA sequence using a sliding window of 30 nucleotides (nt), moving from the start codon to the 120^th ^downstream nucleotide in steps of 10 nt (for a total of 13 windows). We calculated Δ*G *using the RNAfold program in the Vienna package [24,25] under default settings: folding occurred at 37°C; GU pairs were allowed; unpaired bases could participate in at most one dangling end; energy parameters were obtained from [26]. We evaluated only the minimum-free-energy structure.

To quantify the deviation from expectation given a gene's amino-acid sequence and codon usage bias, we also calculated Δ*G *for 1000 permuted mRNA sequences. We obtained permuted sequences by randomly reshuffling synonymous codons within each gene. We then calculated a *Z*-score, *Z*_Δ*G*_, by comparing the Δ*G *of the real mRNA segment to the distribution of Δ*G *values of the permuted sequences, as described [15]. *Z*_Δ*G *_measures the extent to which local mRNA stability deviates from expectation. A positive *Z*_Δ*G *_means that local mRNA stability is reduced, and a negative *Z*_Δ*G *_means that it is increased. We also evaluated the difference in local GC composition between the actual and randomized sequences via a *Z*-score *Z*_GC_, as described [15]. For a few select virus species, we also tested whether our results were confounded by dinucleotide frequencies. We randomized virus mRNA sequences using a dinucleotide shuffling algorithm [27]. This algorithm preserves the dinucleotide composition as well as the codon use frequency in the reshuffled sequence.

We corrected for phylogenetic relationship among viruses by calculating phylogenetically independent contrasts (PIC), using the R library ape, version 2.5-1. We used three separate phylogenetic trees, one for viruses infecting eukaryotes, one for viruses infecting prokaryotes, and one for viruses infecting archaea. Since widely diverged viruses cannot be aligned, the phylogenetic trees we used were constructed purely based on taxonomic classification, as provided by NCBI's taxonomy tool http://www.ncbi.nlm.nih.gov/taxonomy.

We matched viruses to hosts using the "host" attribute in the "source" feature of the genbank file provided by RefSeq. We obtained quantities for hosts (such as GC content, *Z*_Δ*G*_) from our previous study [15].

We carried out all statistical analyses using R, version 2.10.1. Our R scripts plus accompanying raw data files are provided as supplementary data [Additional Files 2 and 3].

## Authors' contributions

TZ and COW designed the study, carried out analyses, prepared figures, and wrote the manuscript. Both authors read and approved the final manuscript.

## Supplementary Material

Additional file 1 **Supplementary Figures**. A single pdf file containing Supplementary Figures S1-S8. Click here for file

Additional file 2 **Supplementary Data Part 1**. A zip file containing raw data plus R scripts to reproduce all analyses. Click here for file

Additional file 3 **Supplementary Data Part 2**. A zip file containing additional raw data used by the R scripts in Additional File 2. Click here for file
