# Supplementary material for: Reduced stability of mRNA secondary structure near the translation-initiation site in dsDNA viruses
Source: BMC Evol Biol. 2011 Mar 7;11:59. doi: 10.1186/1471-2148-11-59 (PMC3072339; doi:10.1186/1471-2148-11-59)
Supplement: Additional file 1 — Supplementary Figures. A single pdf file containing Supplementary Figures S1-S8. [file 1471-2148-11-59-S1.PDF]

Supplementary material for Zhou and Wilke, Reduced stability of mRNA secondary structure near the translation-initiation site in dsDNA viruses

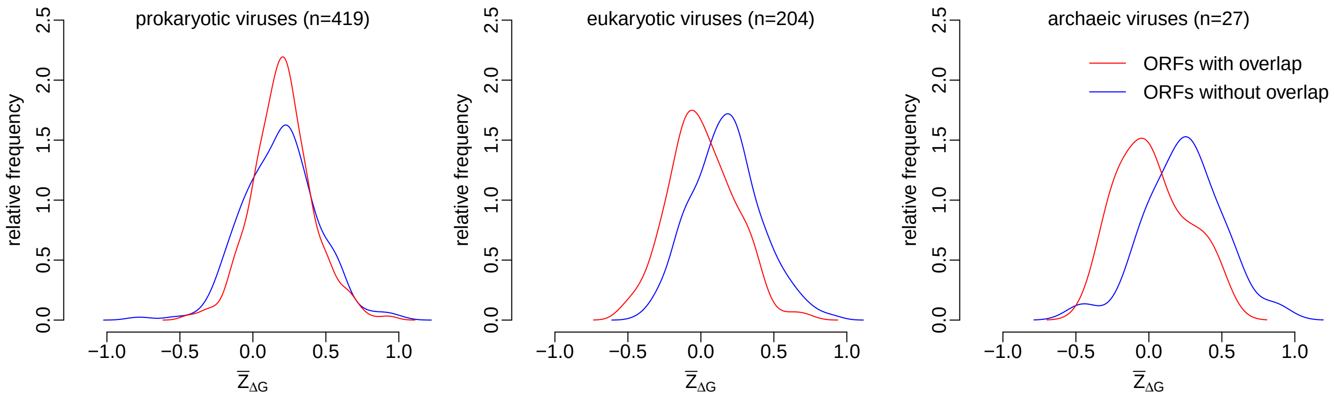

Figure S1: Distributions of $\bar{Z}_{\Delta G}$ in the first window for overlapping and non-overlapping reading frames. For each virus, we averaged $Z_{\Delta G}$ for all overlapping and all non-overlapping reading frames. We then estimated the distributions of these averages using kernel density estimation. For prokaryotic viruses, there is no detectable difference between the distributions (Wilcoxon rank-sum test, $P = 0.38$) while for the other two classes of viruses, the $\bar{Z}_{\Delta G}$ for ORFs with overlap seems to be shifted slightly towards smaller values (Wilcoxon rank-sum test, $P < 10^{-10}$ for eukaryotic viruses and $P = 0.0033$ for archaeic viruses).

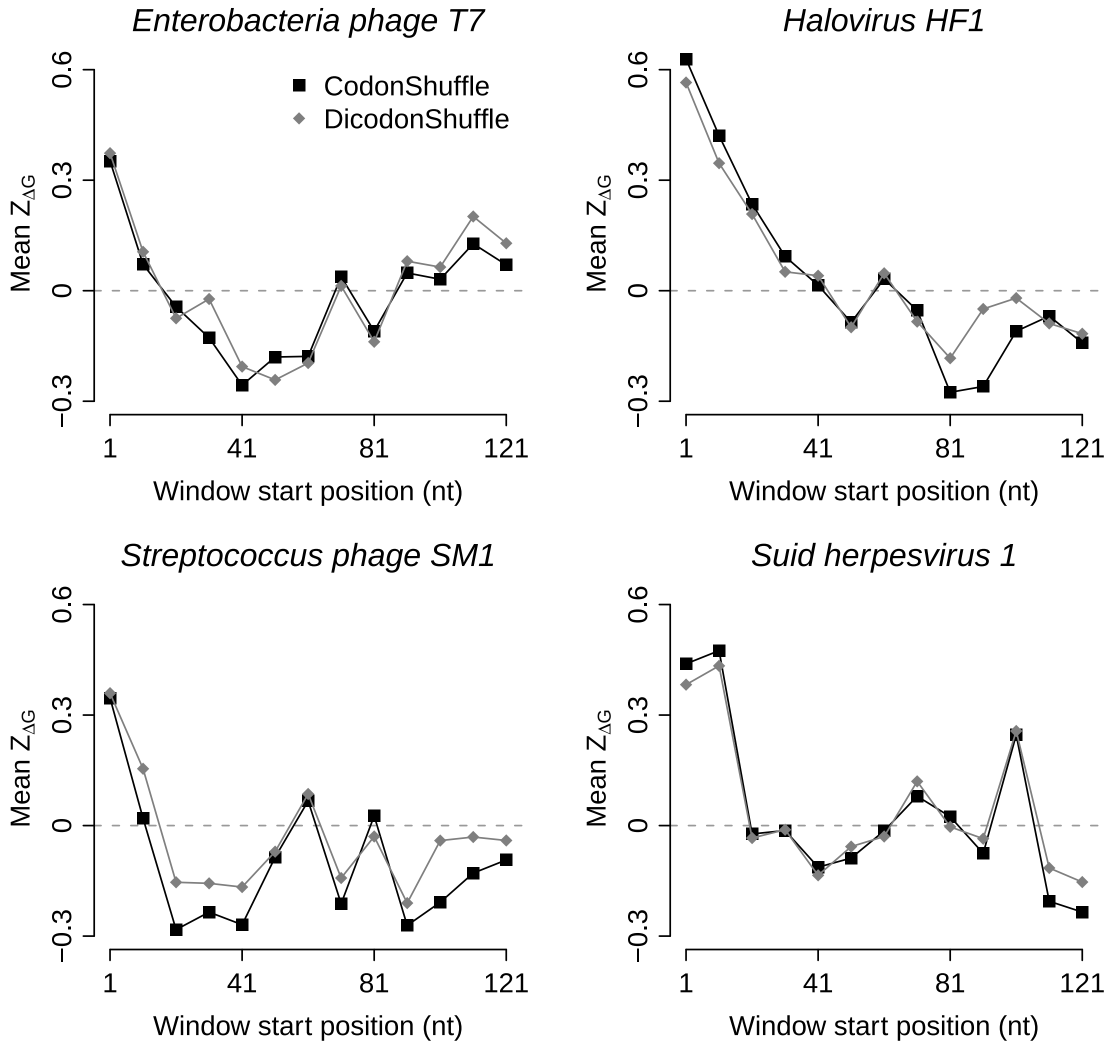

Figure S2: Comparison of the mean $Z_{\Delta G}$ calculated according to the standard codon suffling and the dicodon shuffling, for four representative virus genomes.

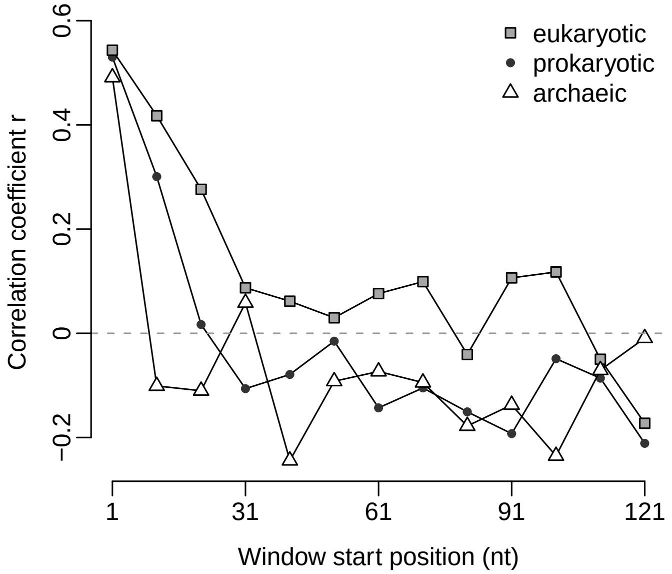

Figure S3: Correlation coefficient r between the PIC of $\bar{Z}_{\Delta G}$ and the PIC of the genomic GC content for dsDNA viruses, as a function of the window position.

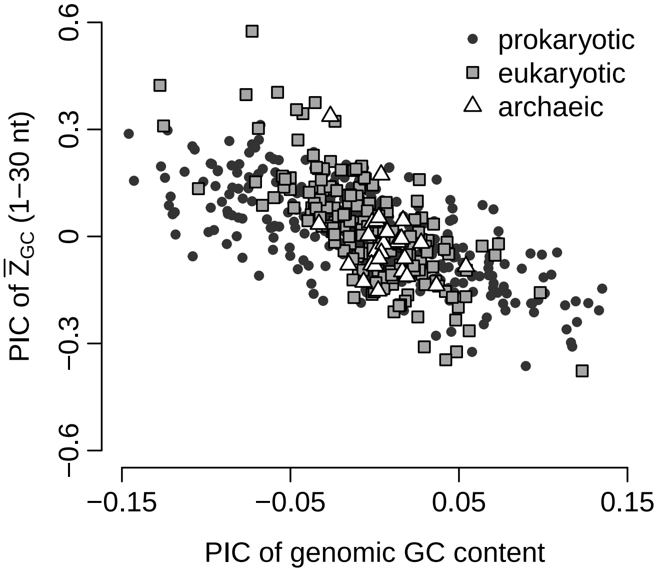

Figure S4: PIC of $\bar{Z}_{\text{GC}}$ of the first window versus PIC of the genomic GC content.

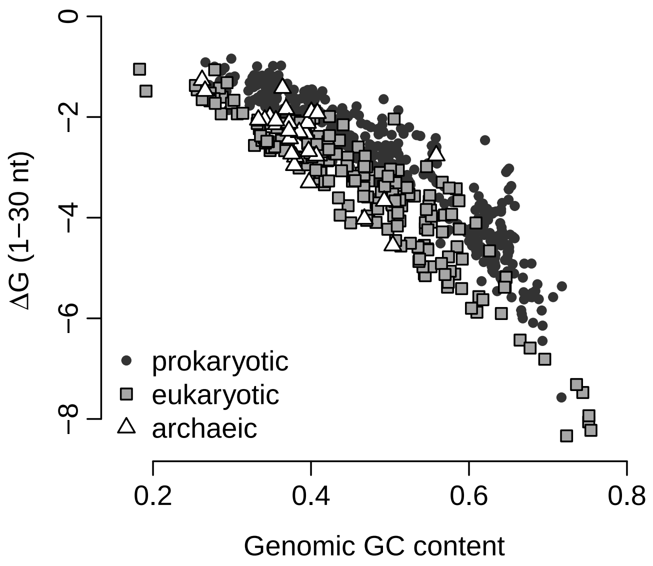

Figure S5: Mean ΔG in the first window versus GC content of viruses.

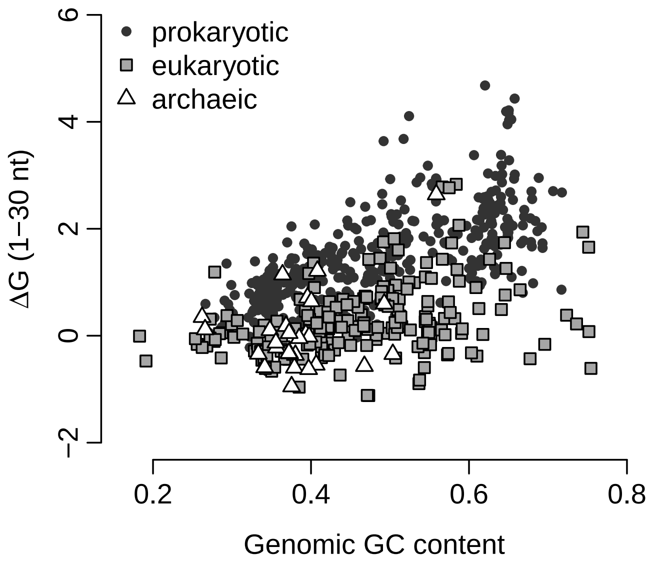

Figure S6: Difference in mean ΔG between the first and the tenth window versus GC content of viruses.

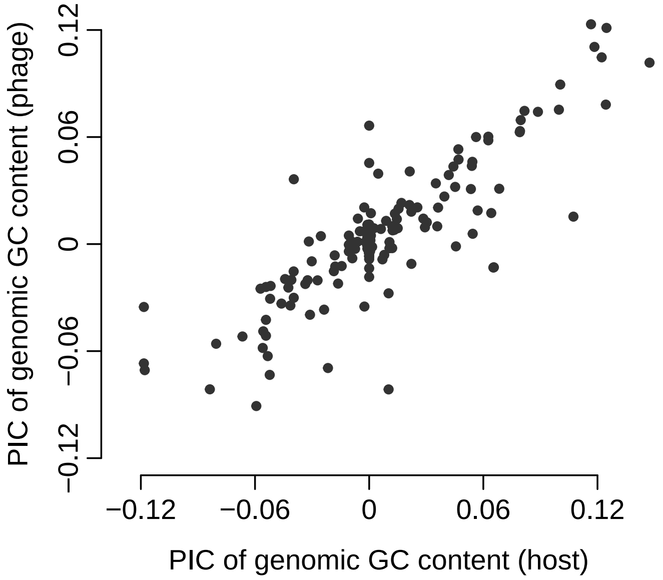

Figure S7: PIC of the genomic GC content in bacteriophages versus PIC of the genomic GC content in their associated hosts. The same phylogenetic tree was used to calculate both sets of PIC.

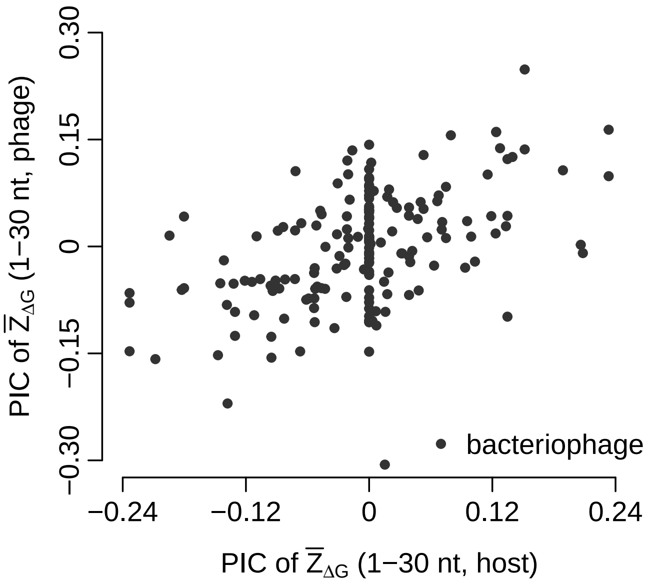

Figure S8: PIC of the 5' $\bar{Z}_{\Delta G}$ in bacteriophages versus PIC of the 5' $\bar{Z}_{\Delta G}$ in their associated hosts. The same phylogenetic tree was used to calculate both sets of PIC.
