# Supplementary material for: Reduced stability of mRNA secondary structure near the translation-initiation site in dsDNA viruses
Source: BMC Evol Biol. 2011 Mar 7;11:59. doi: 10.1186/1471-2148-11-59 (PMC3072339; doi:10.1186/1471-2148-11-59)
Supplement: Additional file 2 — Supplementary Data Part 1. A zip file containing raw data plus R scripts to reproduce all analyses. [file 1471-2148-11-59-S2.ZIP › figures/correlation-zdg-gc.pdf]

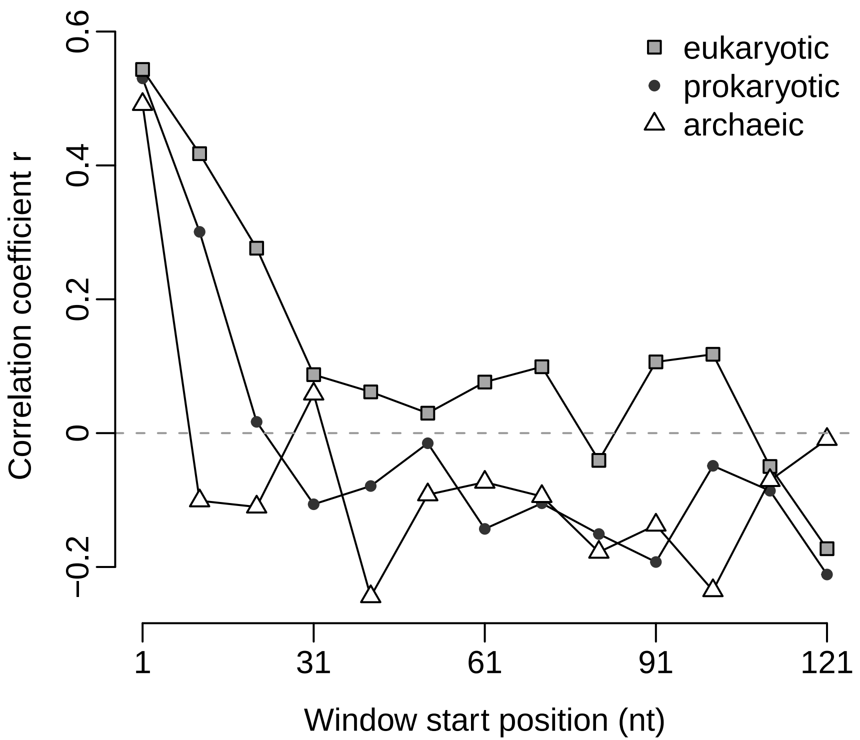

0.6
0.4
0.2
0
−0.2
Correlation coefficient r
eukaryotic
prokaryotic
archaeic
1
31
61
91
121
Window start position (nt)
