# Supplementary material for: Reduced stability of mRNA secondary structure near the translation-initiation site in dsDNA viruses
Source: BMC Evol Biol. 2011 Mar 7;11:59. doi: 10.1186/1471-2148-11-59 (PMC3072339; doi:10.1186/1471-2148-11-59)
Supplement: Additional file 2 — Supplementary Data Part 1. A zip file containing raw data plus R scripts to reproduce all analyses. [file 1471-2148-11-59-S2.ZIP › figures/Zdg_distribution.pdf]

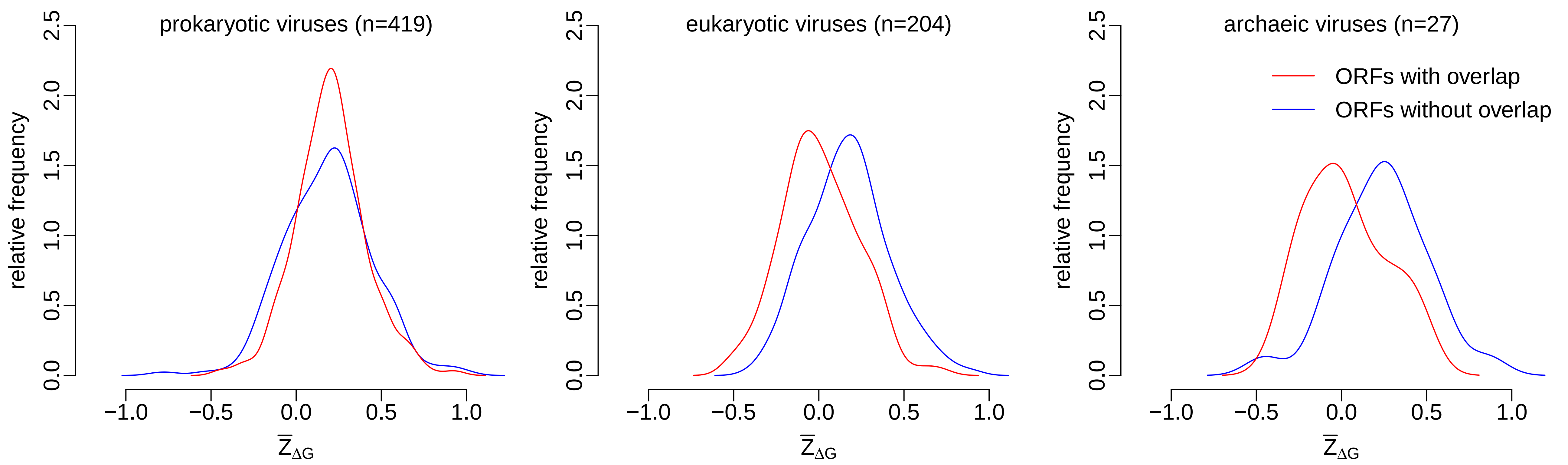
prokaryotic viruses (n=419)
eukaryotic viruses (n=204)
archaeic viruses (n=27)
ORFs with overlap
ORFs without overlap
relative frequency
0.0
0.5
1.0
1.5
2.0
2.5
−1.0
−0.5
0.0
0.5
1.0
$\overline{Z}_{\Delta G}$
