# Supplementary material for: Reduced stability of mRNA secondary structure near the translation-initiation site in dsDNA viruses
Source: BMC Evol Biol. 2011 Mar 7;11:59. doi: 10.1186/1471-2148-11-59 (PMC3072339; doi:10.1186/1471-2148-11-59)
Supplement: Additional file 2 — Supplementary Data Part 1. A zip file containing raw data plus R scripts to reproduce all analyses. [file 1471-2148-11-59-S2.ZIP › figures/pic-zdg1-gc.pdf]

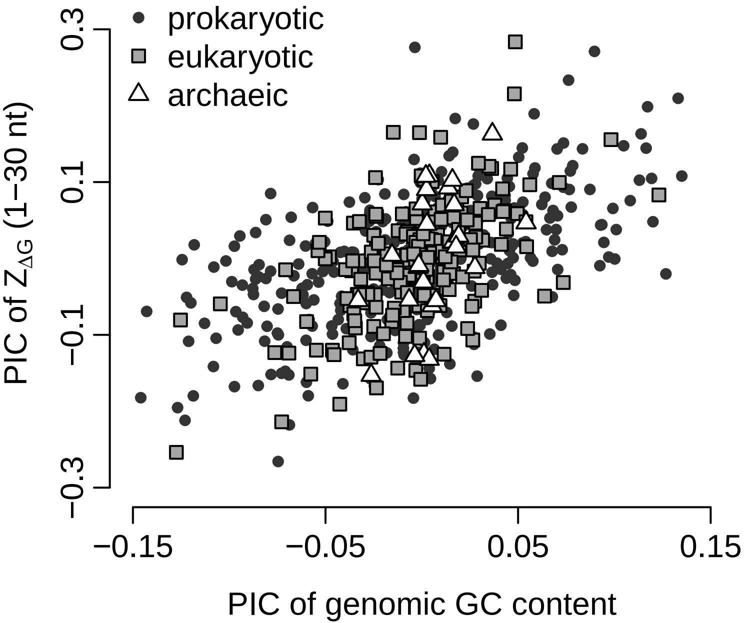

prokaryotic
eukaryotic
archaeic
PIC of $\overline{Z}_{\Delta G}$ (1–30 nt)
0.3
0.1
−0.1
−0.3
−0.15
−0.05
0.05
0.15
PIC of genomic GC content
