# Supplementary material for: Reduced stability of mRNA secondary structure near the translation-initiation site in dsDNA viruses
Source: BMC Evol Biol. 2011 Mar 7;11:59. doi: 10.1186/1471-2148-11-59 (PMC3072339; doi:10.1186/1471-2148-11-59)
Supplement: Additional file 2 — Supplementary Data Part 1. A zip file containing raw data plus R scripts to reproduce all analyses. [file 1471-2148-11-59-S2.ZIP › figures/pic-gc-virus-host.pdf]

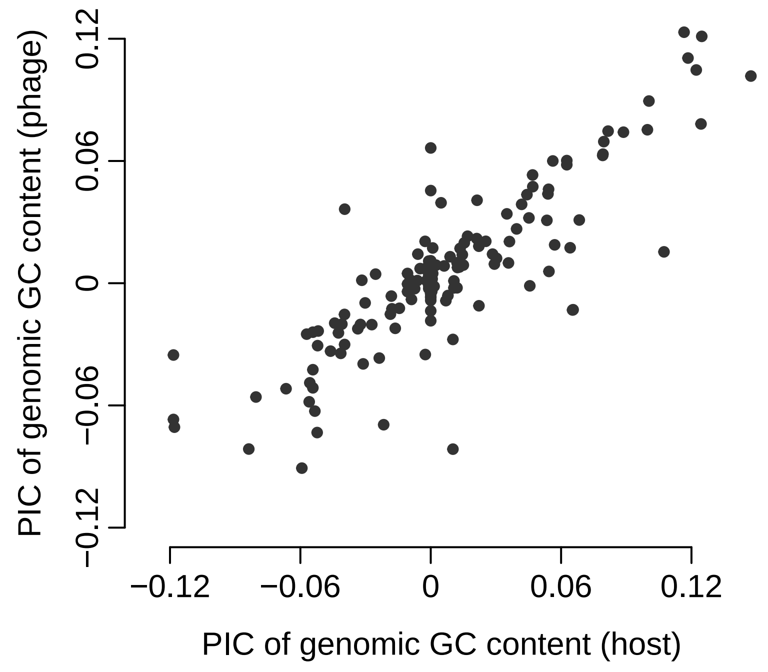

PIC of genomic GC content (phage)
0.12
0.06
0
−0.06
−0.12
−0.12
−0.06
0
0.06
0.12
PIC of genomic GC content (host)
