# Supplementary material for: Reduced stability of mRNA secondary structure near the translation-initiation site in dsDNA viruses
Source: BMC Evol Biol. 2011 Mar 7;11:59. doi: 10.1186/1471-2148-11-59 (PMC3072339; doi:10.1186/1471-2148-11-59)
Supplement: Additional file 2 — Supplementary Data Part 1. A zip file containing raw data plus R scripts to reproduce all analyses. [file 1471-2148-11-59-S2.ZIP › figures/species_counts_by_type.pdf]

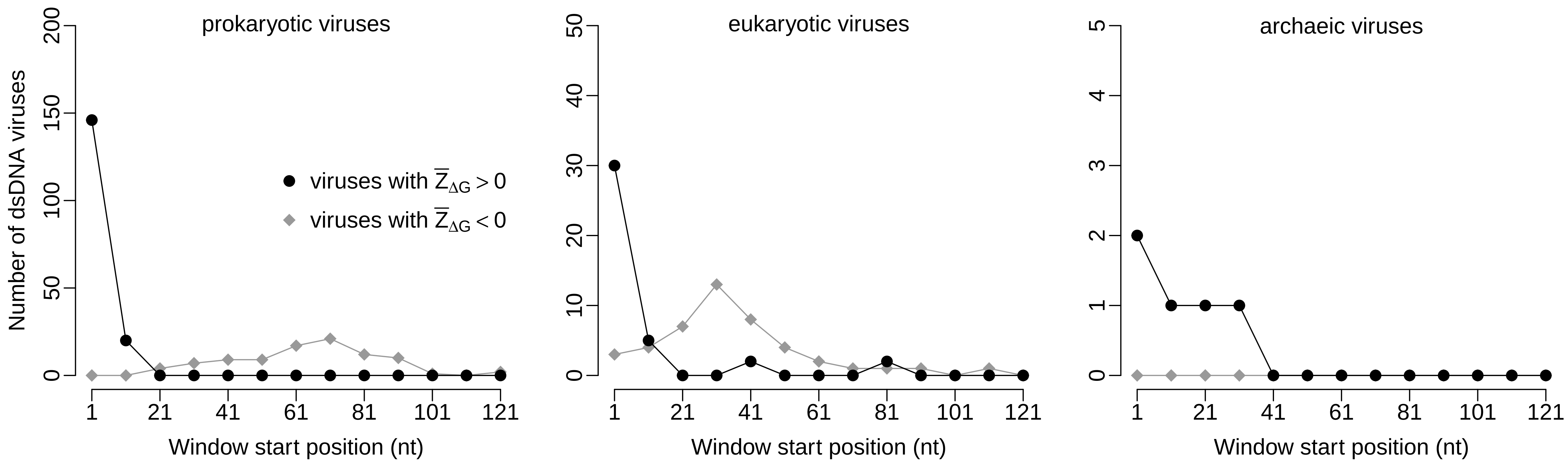

prokaryotic viruses
eukaryotic viruses
archaeic viruses
Number of dsDNA viruses
viruses with $\overline{Z}_{\Delta G} > 0$
viruses with $\overline{Z}_{\Delta G} < 0$
Window start position (nt)
Window start position (nt)
Window start position (nt)
