# Supplementary material for: Reduced stability of mRNA secondary structure near the translation-initiation site in dsDNA viruses
Source: BMC Evol Biol. 2011 Mar 7;11:59. doi: 10.1186/1471-2148-11-59 (PMC3072339; doi:10.1186/1471-2148-11-59)
Supplement: Additional file 2 — Supplementary Data Part 1. A zip file containing raw data plus R scripts to reproduce all analyses. [file 1471-2148-11-59-S2.ZIP › figures/dicodon_shuffle.pdf]

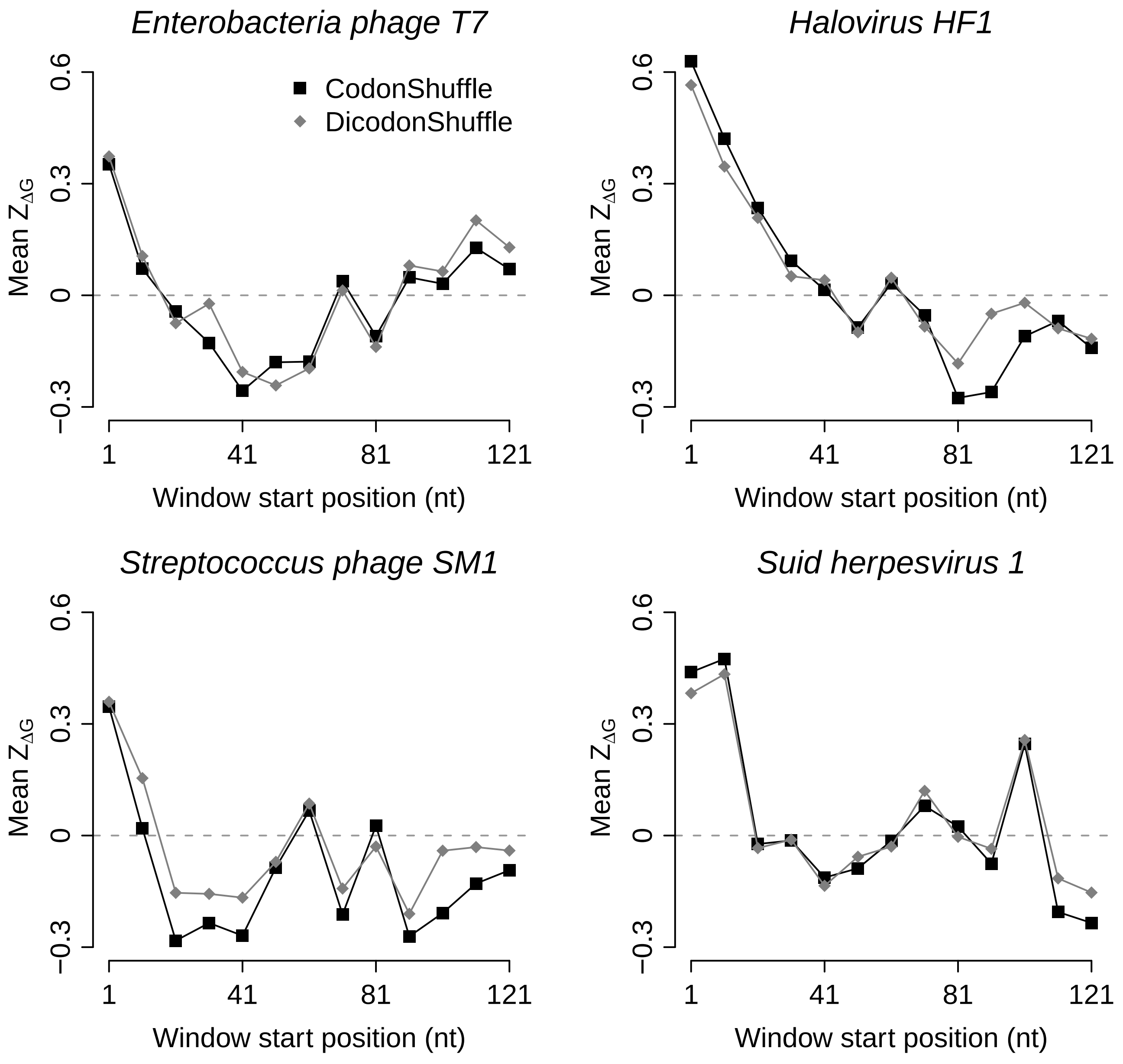

Enterobacteria phage T7
Halovirus HF1
Streptococcus phage SM1
Suid herpesvirus 1
CodonShuffle
DicodonShuffle
Mean Z∆G
Window start position (nt)
0.6
0.3
0
−0.3
1
41
81
121
